# Salt-inducible kinase 2 (SIK2) inhibitor ARN-3236 attenuates bleomycin-induced pulmonary fibrosis in mice

**DOI:** 10.1186/s12890-022-01940-0

**Published:** 2022-04-11

**Authors:** Liangneng Zou, Dequn Hong, Kecong Li, Bingyuan Jiang

**Affiliations:** 1 Department of General Medicine, The Fifth Hospital of Xiamen, 101 Min’an Road, Maxiang, Xiang’an, Xiamen, 361101 Fujian People’s Republic of China; 2 Department of Emergency, The Fifth Hospital of Xiamen, 101 Min’an Road, Maxiang, Xiang’an, Xiamen, 361101 Fujian People’s Republic of China; 3 The Affiliated Second Hospital of Xiamen Medical College, 566 Shengguang Road, Ji’mei, Xiamen, 361000 Fujian People’s Republic of China; 4 Critical Care Medicine, The Fifth Hospital of Xiamen, 101 Min’an Road, Maxiang, Xiang’an, Xiamen, 361101 Fujian People’s Republic of China

**Keywords:** Salt-inducible kinase 2 (SIK2), ARN-3236, cAMP response element binding protein (CREB), CREB-regulated transcription co-activator 2 (CRTC2), Pulmonary fibrosis

## Abstract

**Background:**

Pulmonary fibrosis is a fatal lung disease with complex pathogenesis and limited effective therapies. Salt-inducible kinase 2 (SIK2) is a kinase that phosphorylates CRTCs and regulates many physiological processes. However, the role of SIK2 on pulmonary fibrosis remains unclear, and whether SIK2 inhibitor can attenuate pulmonary fibrosis is unknown.

**Method:**

We subjected human fetal lung fibroblasts (HFLs) to transforming growth factor-β1 (5 ng/mL) for 12 h, and examined the expression of SIK2, CRTCs and pCRTCs in fibroblasts by western-blot. To address the roles of SIK2 and CRTCs involved in the progression of pulmonary fibrosis, HFLs were treated with a small-molecule inhibitor ARN-3236 or by siRNA-mediated knockdown of SIK2 expression. Pulmonary fibrosis model was established with mice by exposing to bleomycin, and assessed by H&E and Masson’s trichrome staining. COL1A and α-SMA distributions were detected in lung tissues by immunohistochemical staining.

**Results:**

We discovered that SIK2 and phosphorylated-CRTC2 were expressed at a low basal level in normal lung tissues and quiescent fibroblasts, but increased in fibrotic lung tissues and activated fibroblasts. Inhibition of SIK2 by ARN-3236 prevented the fibroblasts differentiation and extracellular matrix expression in HFLs and attenuated bleomycin-induced pulmonary fibrosis in mice. Mechanistically, inactivation of SIK2 resulted in the dephosphorylation and nuclear translocation of CRTC2. Within the nucleus, CRTC2 binds to CREB, promoting CREB-dependent anti-fibrotic actions.

**Conclusion:**

In conclusion, our results elucidated a previously unexplored role of SIK2 in pulmonary fibrosis, and identified SIK2 as a new target for anti-fibrosis medicines.

## Introduction

Pulmonary fibrosis or its more severe form idiopathic pulmonary fibrosis (IPF), is a fatal lung disease characterized by alveolar barrier damage, fibroblasts differentiation and excessive deposition of extracellular matrix (ECM) components [1, 2]. The median survival of IPF patients is only 3 years and its incidence and mortality increase with age [1]. External insults, such as environmental insults (e.g., asbestos), autoimmune diseases (e.g., lupus erythematosus) and drugs [e.g., bleomycin (BLM)], can cause the disturbed production of pro-fibrotic mediators, including connective tissue growth factor (CTGF), transforming growth factor-β1 (TGF-β1) and platelet derived growth factor (PDGF) [2–5]. These pro-fibrotic mediators trigger the activation of quiescent fibroblasts and differentiation of fibroblasts to myofibroblasts, resulting in hyperproliferation of fibroblasts and excessive deposition of ECM [2]. Therefore, suppression of fibroblast differentiation and ECM deposition represent a promising therapeutic strategy for protecting pulmonary fibrosis.

The most common medical therapy in use for pulmonary fibrosis is immunosuppressive agents, including corticosteroids, azathioprine and cyclophosphamide. However, their prolonged use is associated with undesirable side-effects, such as osteoporosis, aseptic joint necrosis, neutropenia, renal toxicity and cardiovascular injury. Recently, pirfenidone and nintedanib are approved for the treatment of pulmonary fibrosis for patients with mild to moderate disease. However, the adverse effects, including gastrointestinal side-effects, rash and photosensitivity, limited the efficacy of these drugs in protecting against pulmonary fibrosis. New therapeutic approaches that could attenuate pulmonary fibrosis are still highly desired in both academia and industry.

Salt-inducible kinase 2 (SIK2), a member of the AMP-activated protein kinases (AMPKs) family, is a serine/threonine kinase that phosphorylates the cAMP response element binding protein (CREB)-regulated transcription co-activators (CRTCs) [6, 7]. Phosphorylation by SIK2 promotes the cytoplasm translocation of CRTCs, preventing them from activating the transcription factors CREB [6]. In contrast, inactivation of SIK2 leads to the dephosphorylation and nuclear translocation of CRTCs [6]. Within the nucleus, CRTCs interact with CREB and promote CREB-dependent gene transcription [6, 8, 9]. SIK2 is widely expressed in many tissues and regulates many physiological processes, including innate immunity, bone formation, depression and metabolism [7]. So far, the function of SIK2 under pulmonary fibrosis conditions remains undefined. Interestingly, Dasatinib, a dual tyrosine kinase/SIK2 inhibitor, has been identified as an anti-fibrotic agent in mice with pulmonary fibrosis [10, 11], suggesting that SIK2 inhibition might be an effective anti-fibrosis therapeutic approach.

In the current study, we examined whether inactivation of SIK2 could attenuate pulmonary fibrosis. Our results demonstrated that SIK2 and phosphorylated-CRTC2 were increased in fibrotic lung tissues and activated fibroblast. Inhibition of SIK2 significantly attenuated bleomycin-induced pulmonary fibrosis by promoting dephosphorylation and nuclear translocation of CRTC2 and consequently activating the CREB anti-fibrotic pathway.

## Materials and methods

### Materials

Unless otherwise noted, all reagents were purchased from Sinopharm. SIK2 inhibitor ARN-3236 was purchased from Medchemexpress (HY-120856) and Tsbiochem (T5993). pCREB inhibitor 666-15 was purchased from Medchemexpress (HY-101120) [12], Tsbiochem (T5318) and Cayman Chem (30780).

### Cell culture

Human fetal lung fibroblasts (HFLs) were purchased from the Cell Bank of the Chinese Academy of Sciences (GNHu28). Cells were cultured in Dulbecco’s modified Eagle’s (DMEM) medium supplemented with 10% fetal bovine serum (FBS) (Invitrogen, 10270-106), benzylpenicillin (100 U/mL) and streptomycin (100 µg/mL) in an incubator at 37 °C with 5% CO_2_ atmosphere. Cells in passage 5–10 were used in all experiments [13].

The fibroblasts were seeded into 6-well plates at a density of 5 × 10^5^ per well and cultured until 80% confluence. Cells were then incubated with vehicle (0.1% DMSO), ARN-3236 (0.5 μM), or CREB inhibitor 666-15 (0.5 μM) for 30 min, or silencer select siRNA against SIK2 (Thermo Fisher Scientific, s23355, 100 pM) and HiPerfect transfection reagent (Qiagen, 301704, USA) for 18 h before challenged by vehicle (PBS) or 5 ng/mL TGF-β1. After 12 h, cells were harvested for PCR and western blot analysis [13].

### Human fetal lung fibroblasts (HFLs) overexpressing SIK2 (HFLs-SIK2)

HFLs-SIK2 cells were constructed using a previously reported method [14]. The cDNA fragments of human SIK2 were cloned into the lentiviral expression vector pCDH-CMV-MCS-EF1α-Puro (System Biosciences, D510) to obtain the SIK2-overexpressing plasmid pCDH-SIK2. The recombinant plasmid pCDH-SIK2, lentivirus packaging plasmids (pMD2.G and psPAX2) were co-transfected into HEK293T cells. Two days after incubation, culture supernatants were collected and filtrated through filters (Invitrogen, LC2003, 0.45 µm) to obtain the replication defective virus, following by transducing into HFLs supplemented with polybrene (Sigma, TR-1003, 8 μg/mL). The stable HFLs-SIK2 cells were obtained by puromycin selection at a concentration of 10 μg/mL.

### Cell proliferation assay

Cells were seeded into 96-well plates at a density of 5 × 10^3^ per well and cultured for 12 h. Cells were then incubated with vehicle (0.1% DMSO), ARN-3236 (0.5 μM), or CREB inhibitor 666–15 (0.5 μM) for 30 min, or silencer select siRNA against SIK2 (Thermo Fisher Scientific, s23355, 100 pM) and HiPerfect transfection reagent (Qiagen, 301704, USA) for 18 h before challenged by vehicle (PBS) or 5 ng/mL TGF-β1 for 12 h. The 3-(4,5-Dimethylthiazol-2-yl)-2,5-diphenyltetrazolium bromide (MTT) (Aladdin, D274386, 5 mg/mL, 10 μL) solution was added into each well, and incubated at 37 °C for 4 h. The supernatant was then removed and DMSO (150 μL) was added. After 10 min of incubation at 37 °C, the absorbance of samples was measured by a microplate reader at a wavelength of 490 nm for 3 min. Percent cell proliferation was defined as the relative percentage (%) of treated cells relative to untreated control group [13].

### Western blot

Proteins were extracted from HFLs using RIPA lysis buffer (Beyotime, P0013B) and quantified by the BCA protein assay kit (Beyotime, P0012S). After separation by 10% sodium dodecyl sulfate (SDS)–polyacrylamide gel electrophoresis, the proteins were transferred onto a Hybond-P membrane (Amersham Biosciences). The membrane was blocked with 8% skim milk powder in TBST for 1 h at 25 °C, and then incubated with rabbit anti-human pCRTC1 antibody (Sigma, ABE560, dilution 1:1000), rabbit anti-human pCRTC2 antibody (Abcam, ab109081, dilution 1: 1000), rabbit anti-human CRTC2 antibody (Cell Signaling Technology, 9198, dilution 1: 1000), rabbit anti-human pCREB antibody (Abcam, ab32096, dilution 1: 1000) and GADPH (Cell Signaling Technology, 5174, dilution 1: 2000) at 4 °C for 12 h. After extensive washing with 1% TBST, the membrane was incubated with the horseradish peroxidase-conjugated goat anti-rabbit secondary antibody (Cell Signaling Technology, 7074, dilution 1: 5000) for 1 h at room temperature. Bands were visualized with an enhanced chemiluminescence detection kit (Thermo, WP20005). Quantitative analyses were performed using Image J software, with GADPH as the internal standard [15]. Images of full-length western blots presented in supporting information.

### Animal

All experimental procedures and animal usage were carried out and approved by the Animal Care and Use Committee of Xiamen Medical college (Approval No. FJMU IACUC 2020-0124). Mice were group-housed in ventilated cages at the Animals Housing Unit of Xiamen Medical college with controlled temperature (25 ± 1 °C), relative humidity (55% ± 10%) and 12 h light–dark cycle.

### Immunofluorescence staining

HFLs were cultured on coverslips under the same conditions as described above. Cells were fixed at 4 °C with 4% paraformaldehyde for 30 min, permeabilized with 0.1% Triton X for 15 min at room temperature and then blocked with 1% BSA for 30 min. The cells were then incubated for 12 h at 4 °C with rabbit anti-mouse CRTC2 antibody (Abcam, ab244418, dilution 1:500). After extensive washing with 0.1 M PBS, cells were incubated with goat anti-rabbit IgG 647 (Abcam, ab150079, dilution 1:1000) for 2 h at room temperature, post-fixed and cover slipped with Antifade mounting medium with 4′,6-diamidino-2-phenylindole (DAPI). Immunofluorescence was visualized using a confocal microscope (Olympus, Japan). The translocation rate was counted from all fields [16].

### Bleomycin (BLM)-induced mouse pulmonary fibrosis

Briefly, BALB/C male mice (6–7 weeks old, 18–22 g) were anesthetized by intravenous injection of pentobarbital sodium (35 mg/kg) and then intratracheally administrated with BLM (5 mg/kg, dissolved in 100 μL of PBS) on day 0 [17]. ARN-3236 (10, 30 mg/kg), 666–15 (10 mg/kg) or its vehicle (15% PEG400 and 15% tween 80 saline solution, 1 mL/kg) was intraperitoneally (i.p.) administered once daily starting from the day of BLM application. Mice were sacrificed by CO2 inhalation 14 and 28 days after BLM instillation, and lung tissues were harvested for histological analysis, hydroxyproline content, and western blotting.

### Lung histological analysis

Lungs were harvested, fixed in 4% (w/v) neutral phosphate-buffered paraformaldehyde for 24 h, followed by embedding in paraffin. Lung tissues were cut into 5-μm sections which were stained with hematoxylin–eosin (H&E) or Masson’s trichrome after deparaffinized with xylene, and were photographed using a light microscope (Motic, China) at the magnification of × 100. The severity of fibrosis was then assessed the Ashcroft score [18, 19]. All of the lung sections were scored blindly and independently by at least two independent observers. Total 3 sections per animal were analyzed. The Ashcroft score was obtained from 3 randomly chosen and nonoverlapping fields (400 × 400 µm) in each section.

### Immunochemical staining

Immunohistochemical staining was performed on paraffin embedded lung sections. The sections were deparaffinized with xylene and prepared for staining as described previously. Sections were blocked with 10% goat serum in PBS for 1 h, followed by incubation with the following primary antibodies at 4 °C for 12 h: rabbit anti-mouse α-SMA (Abcam, ab124964, dilution 1:500) and rabbit anti-mouse COL1A (Abcam, ab260043, dilution 1:500). After washing, sections were incubated with biotin-conjugated goat anti-rabbit IgG and avidin–biotin peroxidase complex (DBA) at room temperature for 2 h, and photographed using a light microscope (Motic, China) at the magnification of × 100. To assess nonspecific staining for α-SMA and COLA1, alternate sections from each experimental condition were also incubated with primary or secondary antibody only [20].

### Hydroxyproline assay

The pulmonary hydroxyproline content were measured using a previous reported method [17]. In brief, lung tissue (30 mg) was homogenized in 10 N concentrated NaOH (1 mL), and the mixtures were incubated for 60 min at 95 °C. After cooling to room temperature, the hydrolysates were neutralized to pH 6.0–6.8 with 10 N concentrated HCl, following by centrifugation at 10,000×*g* for 5 min at room temperature. The supernatant was collected, and the hydroxyproline content was evaluated with a hydroxyproline assay kit (Abcam, Ab222941) following the manufacturer’s instructions. The results were expressed as μg hydroxyproline/ mg lung tissue.

### Real-time polymerase chain reaction (RT-PCR)

Total RNA was collected using TRIzol (Invitrogen) according to the manufacturer’s protocol. RNA concentrations were determined by spectrophotometer (Beckman Coulter). A total of 2 μg of RNA were subjected to reverse transcription using the ReverTra Ace qPCR RT Kit (TOYOBO, China) to prepare cDNA according to the manufacturer’s instructions. RT-PCR was performed in a 7300 real-time PCR System (Applied Biosystems, USA) using SYBR Premix Ex Taq GC (Takara, China). Each sample was tested three times. Reaction conditions consisted of one cycle at 95 °C for 30 s (1 cycle), 60 °C for 60 s (1 cycle), and extension at 72 °C for 60 s (35 cycles), and a final melting curve analysis. Primers were as follows:

- *Mouse α-SMA* 5′-ACTGGGACGACATGGAAAAG-3′ (Forward); 5′-CATCTCCAGAGTCCAGCACA-3′ (Reverse).
- *Mouse Collagen 1A (COL1a)* 5′-GAGCGGAGAGTACTGGATCG-3′ (Forward); 5′-TACTCGAACGGGAATCCATC-3′ (Reverse).
- *Mouse TGF-β1* 5′-TGATACGCCTGAGTGGCTGTCT-3′ (Forward); 5′-CACAAGAGCAGTGAGCGCTGAA)-3′ (Reverse).
- *Mouse fibronectin* 5′-GATGTCCGAACAGCTATTTACCA-3′ (Forward); 5′-CCTTGCGACTTCAGCCACT-3′ (Reverse).
- *Mouse GAPDH* 5′-AGTGGCAAAGTGGAGATT-3′ (Forward); 5′-GTGGAGTCATACTGGAACA-3′ (Reverse).
- *Human fibronectin* 5′-AGGACGGACATCTTTGGTGC-3′ (Forward); 5′-TGTGGT TGTTGTATAGGAAGGG-3′ (Reverse).
- *Human Collagen 1A (COL1a)* 5′-CAAGACGAAGACATCCCAC-3′ (Forward); 5′-CGGTTGATTTCTCATCATAGC-3′ (Reverse).
- *Human α-SMA* 5′-AGAGTTACGAGTTGCCTGATGG-3′ (Forward); 5′-GATGCTGTTGTAGGTGGTTTCA (Reverse).
- *Human GADPH* 5′-AGG GCT GCT TTT AAC TCT GGT-3′ (Forward); 5′-CCC CAC TTG ATT TTG GAG GGA-3′ (Reverse).

### Statistical analysis

Data were subjected to statistical analyses with GraphPad Prism 5.0 software and represented as means ± S.E.M. Unpaired Student’s test (*t* test) was used to compare differences between two groups. Three or more different groups were analyzed by one-way analysis of variance (ANOVA) with Dunnett’s post hoc multiple comparison tests. In all cases, a *P* value < 0.05 was considered statistically significant.

## Results

### Inactivation of SIK2 leads to the dephosphorylation and nuclear translocation of CRTC2 in HFLs

**Fig. 1:**
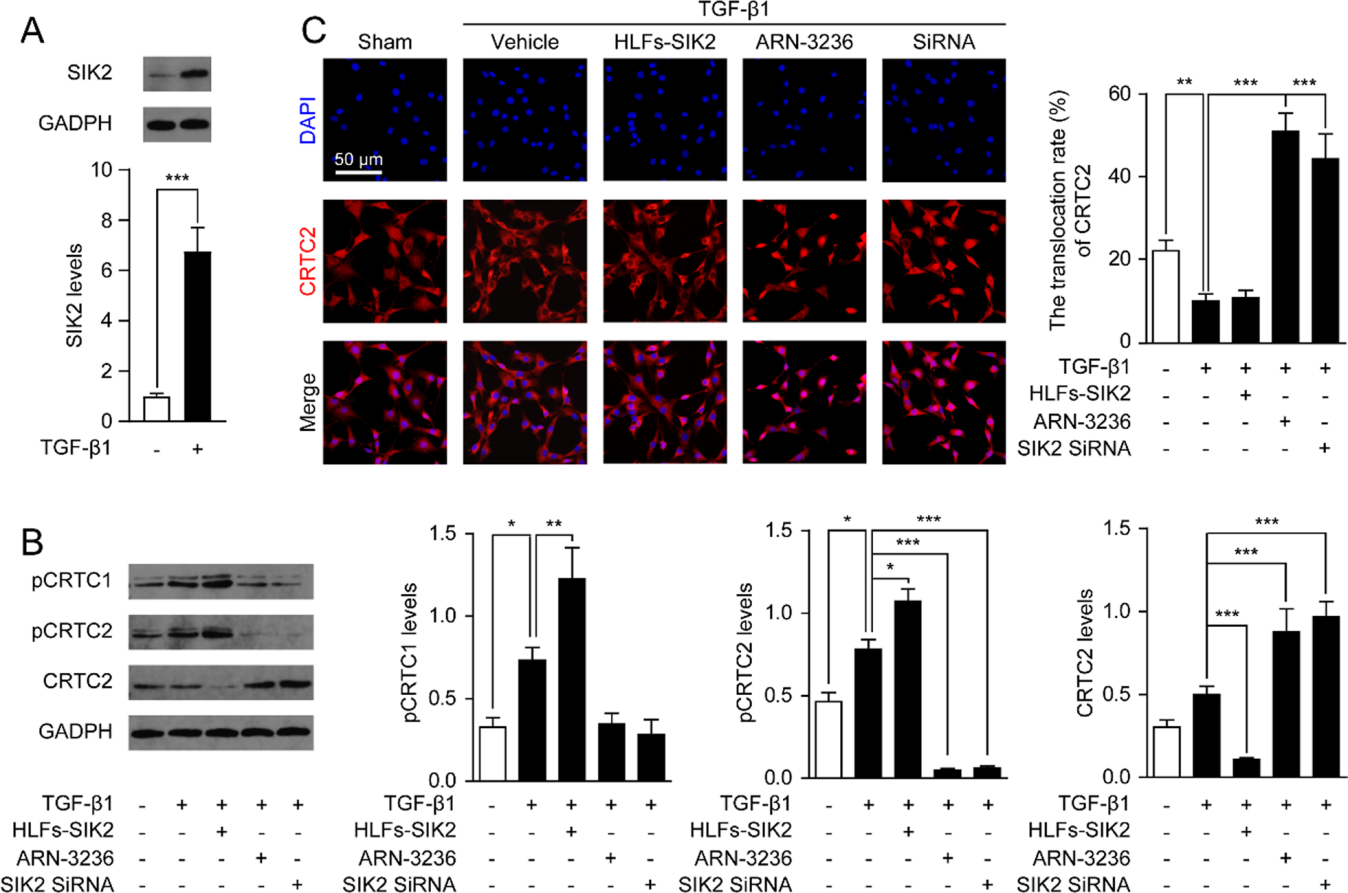
Inactivation of SIK2 led to the dephosphorylation and nuclear translocation of CRTC2 in human fetal lung fibroblasts (HFLs). HFLs or SIK2 overexpressed HFLs (HFLs-SIK2) were treated with DMSO (0.1%), ARN-3236 (0.5 μM) or transfected with SIK2 SiRNA (100 nM). Cells were then treated with TGF-β1 (5 ng/mL) for 12 h. **A**, **B** Representative western blot bands and quantitation of SIK2, pCRTC1, pCRTC2 and CRTC2 protein expressions in HFLs. **C** Representative confocal images and quantification of the nuclear translocated CRTC2 in HFLs and HFLs-SIK2 cells. Data are expressed as mean ± SEM of at least 4 independent experiments. ***P < 0.001; **P < 0.01; *P < 0.05

To address whether SIK2 and CRTCs might be involved in the progression of pulmonary fibrosis. We subjected HFLs to TGF-β1 (5 ng/mL) for 12 h, and examined the expression of SIK2 and CRTCs in fibroblasts. As shown in Fig. 1A, the protein levels of SIK2 were significantly increased in fibrotic cells when compared with the normal fibroblasts. Additionally, the phosphorylation of CRTC1 and CRTC2 were increased in HFLs after TGF-β1 stimulation, and were further enhanced by overexpression of SIK2 in HFLs (Fig. 1B). In contrast, inactivation of SIK2 with a small-molecule inhibitor ARN-3236 or by siRNA-mediated knockdown of SIK2 expression, significantly reduced the phosphorylation of CRTC2 and increased the protein levels of CRTC2 in HFLs treated with TGF-β1 (Fig. 1B). In addition, inactivation of SIK2 had no effects on the phosphorylation of CRTC1 (Fig. 1B). We also examined the location of CRTC2 in TGF-β1 stimulated fibroblasts. CRTC2 were distributed in both cytoplasm and nucleus in the basal state, but translocated to the cytoplasm after stimulation with TGF-β1 (Fig. 1C). ARN-3236 and SIK2 siRNA significantly increased the nuclear translocation of CRTC2 (Fig. 1C). Taken together, these results indicated that SIK2 and the phosphorylation of CRTC2 regulated by SIK2 is involved in the progression of pulmonary fibrosis.

### Inactivation of SIK2 inhibits the TGF-β1-induced fibroblast differentiation and the corresponding ECM expression in vitro

**Fig. 2:**
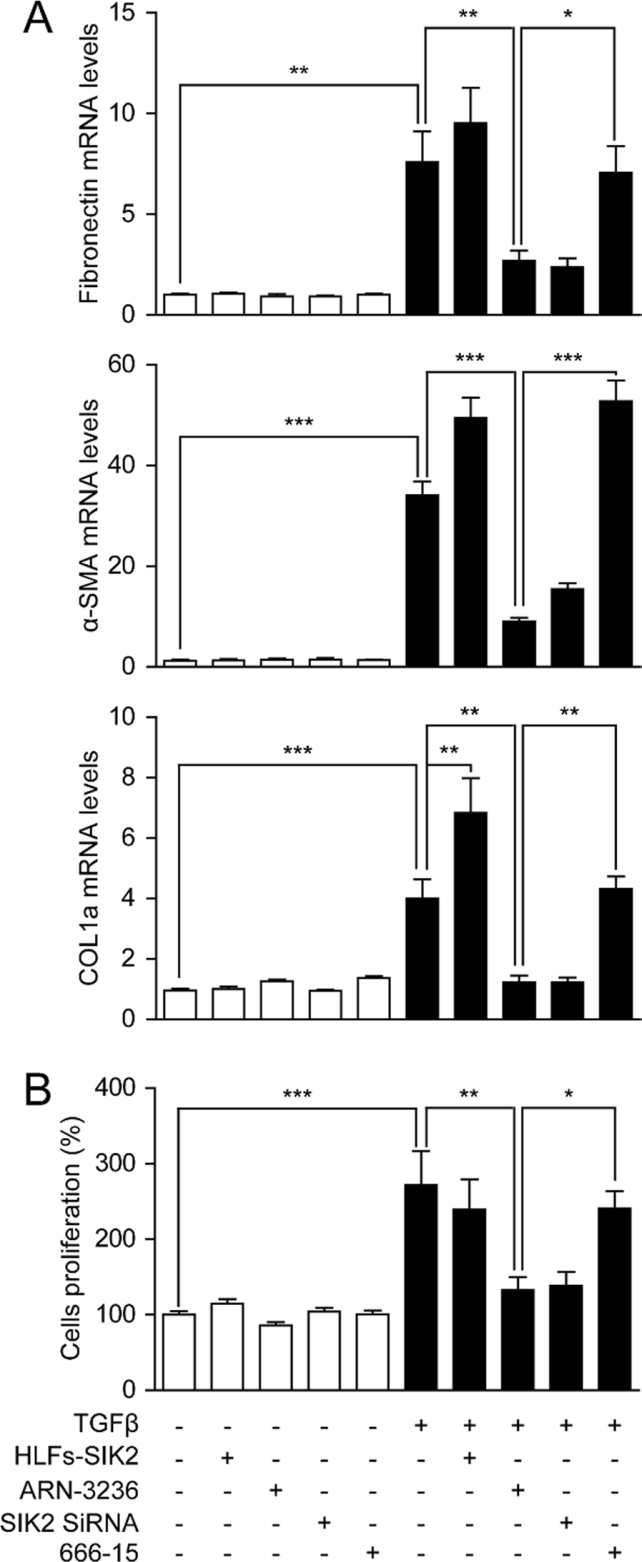
Inactivation of SIK2 inhibited the fibroblast differentiation and TGF-β1 induced pro-fibrotic mediator expression in HFLs. HFLs or SIK2 overexpressed HFLs (HFLs-SIK2) were treated with DMSO (0.1%), ARN-3236 (0.5 μM), 666-15 (0.5 μM) or transfected with SIK2 SiRNA (100 nM). Cells were then treated with TGF-β1 (5 ng/mL) for 12 h. Inactivation of SIK2 inhibited **A** mRNA expression of α-SMA, COL1A and fibronectin and **B** cell proliferation in HFLs. Data are expressed as mean ± SEM of at least 4 independent experiments. ***P < 0.001; **P < 0.01; *P < 0.05

TGF-β1-induced fibroblasts proliferation, differentiation and synthesis of matrix proteins contribute to the pathogenesis of pulmonary fibrosis [5]. Stimulation with TGF-β1, but not its vehicle, triggered the differentiation of fibroblasts characterized by elevated α-SMA levels, a biomarker for myofibroblasts, and the expression of ECM, including fibronectin and collagen type 1A (COL1A) (Fig. 2A). Overexpression of SIK2 in HFLs accelerated fibroblasts differentiation and ECM production, while ARN-3236 and SIK2 siRNA each suppressed these responses (Fig. 2A). Moreover, inactivation of SIK2 significantly suppressed TGF-β1-induced HFLs survival and proliferation (Fig. 2B). Additionally, treatment with 666-15, a potent and selective inhibitor of CREB-mediated gene transcription [12], significantly prevented the anti-fibrotic actions of ARN-3236 (Fig. 2A, B), revealing that SIK2 inactivation may alleviate fibrosis through a CRTC2-mediated CREB activation mechanism.

### Inhibition of SIK2 reduces CRTC2 phosphorylation in lungs.

**Fig. 3:**
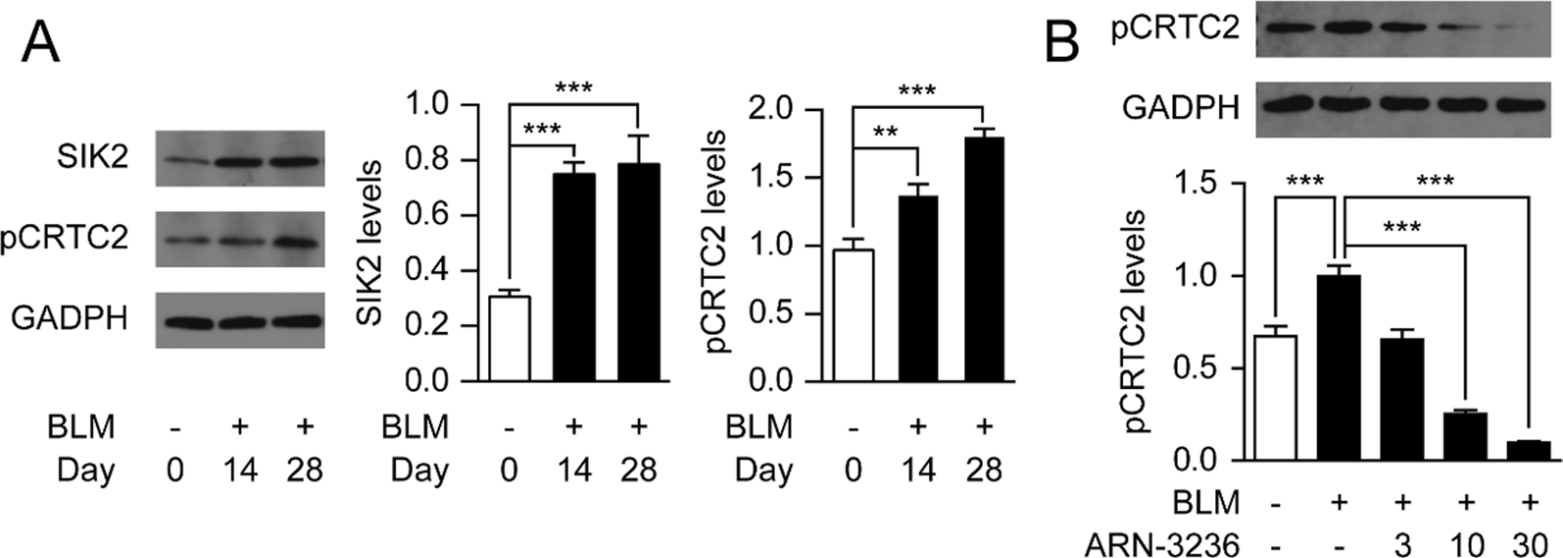
Inhibition of SIK2 reduced CRTC2 phosphorylation in mice. Male BALB/C mice were anesthetized and received intranasal instillation of BLM (5 mg/kg) or its saline vehicle, followed by receiving administration of ARN-3236 (10, 30 mg/kg) or its vehicle. **A** Representative western blot bands and quantitation of SIK2 and pCRTC2 protein expressions in lungs at day 14 and 28. **B** Representative western blot bands and quantitation of pCRTC2 protein expressions in lungs at day 28. Data are expressed as mean ± SEM of at least 6 independent experiments. ***P < 0.001; **P < 0.01; *P < 0.05

Encouraged by the above promising in vitro data, we further examined the role of SIK2 on pulmonary fibrosis in BLM-induced mice model. Consistent with the results found in HFLs, instillation of BLM persistently increased SIK2 levels in lung tissues on day 14 and 28, which were accompanied by elevated phosphorylated-CRTC2 (pCRTC2) content (Fig. 3A). Inhibition of SIK2 by ARN-3236 dose-dependently suppressed the phosphorylation of CRTC2 in lungs (Fig. 3B). These data suggested that ARN-3236 can affect phosphorylation of CRTC2 in vivo.

### Inhibition of SIK2 reduces BLM-induced pulmonary inflammation in mice

**Fig. 4:**
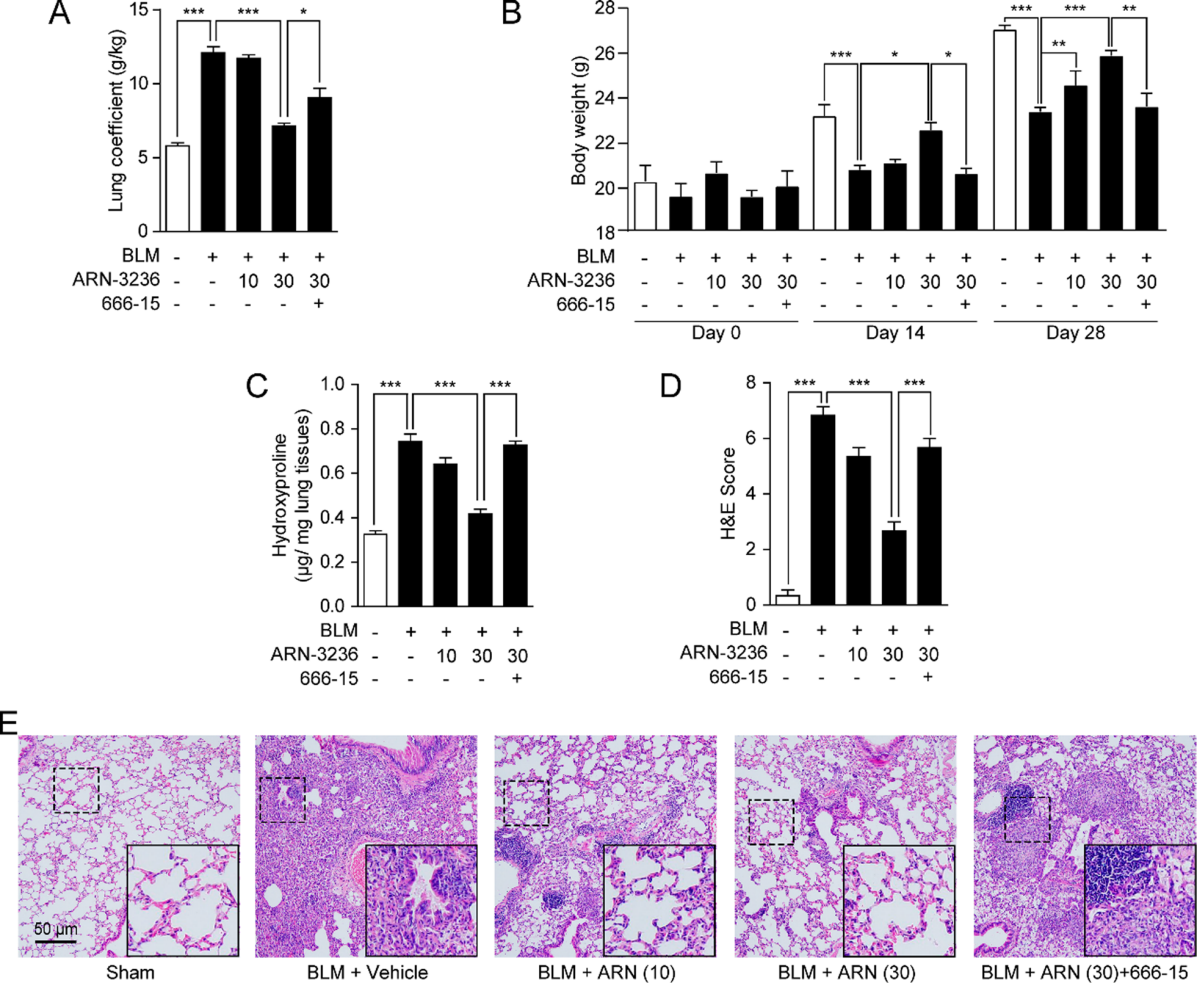
Inhibition of SIK2 attenuates bleomycin (BLM)-induced pulmonary fibrosis in mice. Male BALB/C mice were anesthetized and received intranasal instillation of BLM (5 mg/kg) or its saline vehicle, followed by receiving administration of ARN-3236 (10, 30 mg/kg), CREB inhibitor 666-15 (10 mg/kg) or their vehicle. Lung tissues were isolated at day 28 for analysis. **A** Lung coefficient measured at day 28. **B** Mice body weight and lung coefficient measured at day 0, 14 and 28. **C** The content of hydroxyproline in lung tissues. **D** Semiquantitative analysis of lung tissues by H&E staining. **E** Representative histopathological sections of lung tissues by H&E staining. Data are expressed as mean ± SEM of at least 6 independent experiments. ***P < 0.001; **P < 0.01; *P < 0.05

We further studied the effects of ARN-3236 in BLM-induced pulmonary fibrosis in mice. Instillation of BLM reduced body weight and increased lung coefficient in mice, while that effect was partly reversed after treatment with ARN-3236 (Fig. 4A, B). ARN-3236 also reversed BLM-induced increase in formation of hydroxyproline, a major component of the collagen protein, in lung tissues (Fig. 4C). H&E staining showed that BLM induced disordered morphological structure in the lung, including the fibrotic scarring of lung parenchyma and the thickness of alveolar wall, while inhibition of SIK2 by ARN-3236 reduced the formation of fibrosis foci in the lung (Fig. 4D, E).

**Fig. 5:**
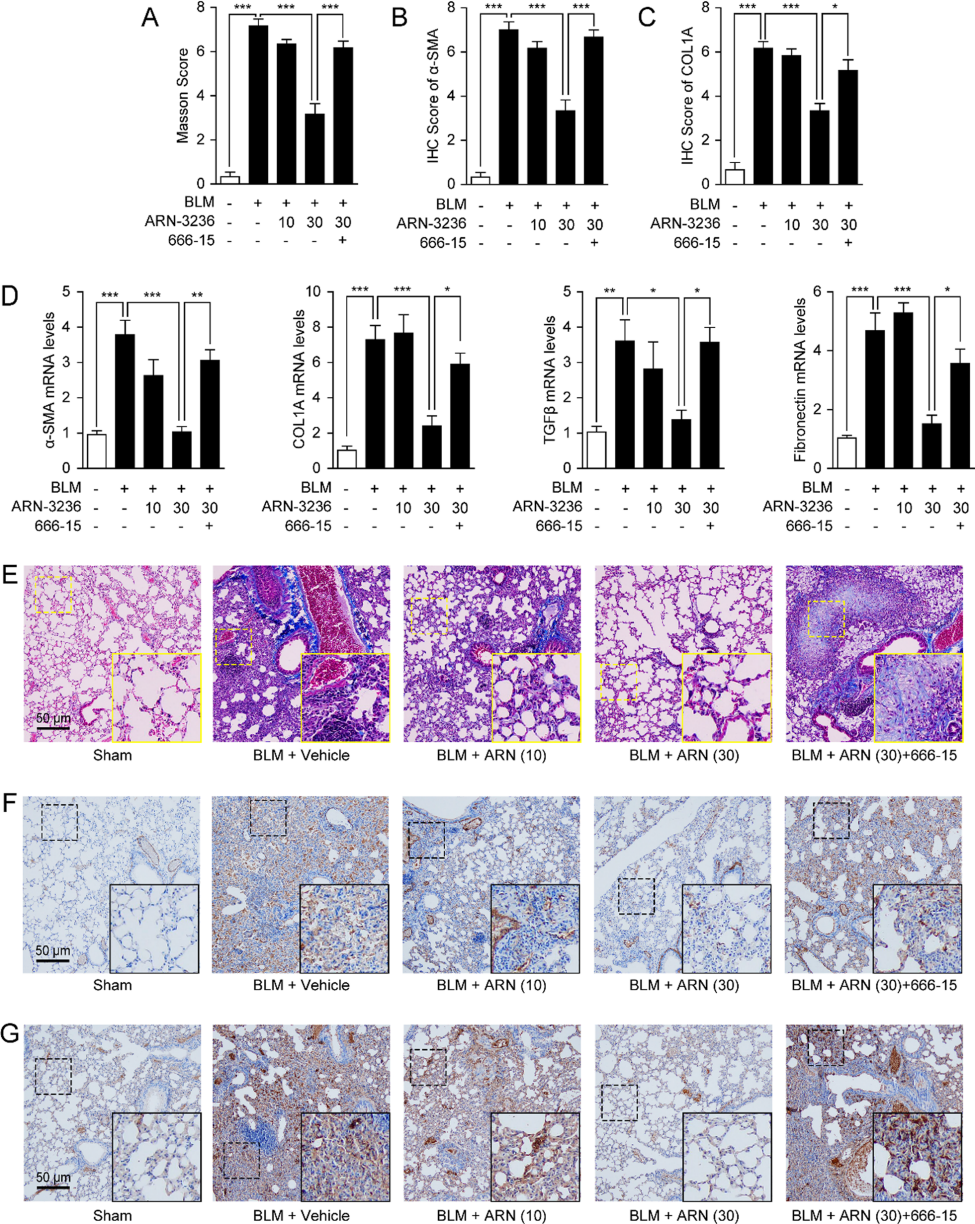
Inhibition of SIK2 attenuates bleomycin (BLM)-induced pulmonary fibrosis in mice. Male BALB/C mice were anesthetized and received intranasal instillation of BLM (5 mg/kg) or its saline vehicle, followed by receiving administration of ARN-3236 (10, 30 mg/kg), CREB inhibitor 666-15 (10 mg/kg) or their vehicle. Lung tissues were isolated at day 28 for analysis. Semiquantitative analysis of **A** Masson’s trichrome staining in **E**, **B** α-SMA expression in **F** and **C** COL1A expression in **G** were scored. **D** mRNA expression of α-SMA, COL1A, TGF-β1 and fibronectin in lungs. **E** Representative histopathological sections and semiquantitative analysis of lung tissues by Masson’s trichrome staining. Representative images of the immunohistochemical **F** α-SMA and **G** COL1A expression in lung tissues. Data are expressed as mean ± SEM of at least 6 independent experiments. ***P < 0.001; **P < 0.01; *P < 0.05

Moreover, Masson’s Trichome staining showed that ARN-3236 reduced BLM-induced dense collagen deposition in lung parenchyma (Fig. 5A, B). Immunochemistry staining also revealed that ARN-3236 reduced α-SMA formation and COL1A accumulation (Fig. 5B, C, F, G). Consistently, treatment with ARN-3236 also suppressed mRNA expression of TGF-β1 and ECM proteins, including α-SMA, fibronectin, and COL1A in fibrotic lungs (Fig. 5D). Furthermore, the anti-fibrotic activity of ARN-3236 was blocked by the CREB inhibitor 666-15, indicating that inactivation of SIK2 might alleviate pulmonary fibrosis through CRTC2-mediated CREB pathway (Figs. 4, 5). When combined, these results suggested that inhibition of SIK2 protected against BLM-induced pulmonary fibrosis in mice.

## Discussion

Pulmonary fibrosis is a progressive and fatal disease with high mortality. Currently, there are no consistently effective approaches to prevent pulmonary fibrosis or slow its progression. For many decades, very limited treatment options were available for patients with pulmonary fibrosis. Effective pharmacologic approaches are still highly desired for pulmonary fibrosis treatment, especially for preventing fibroblast differentiation and ECM deposition. In the present study, we proposed inhibition of SIK2 by its potent inhibitor ARN-3236 as a novel anti-fibrotic approach. We demonstrated the capability of ARN-3236 to prevent fibroblast differentiation and activation, attenuate ECM deposition and reduce immune cell recruitment in BLM induced pulmonary fibrosis. We found that SIK2 was expressed at a low basal level in normal lung tissues and quiescent fibroblasts, but increased in fibrotic lung tissues and activated fibroblast (Figs. 1A, 3A). Inactivation of SIK2 alleviated BLM-induced lung fibrosis in mice and suppressed fibroblast activation and differentiation. Consistently, dasatinib, a dual tyrosine kinase/SIK2 inhibitor, has been identified as an anti-fibrotic agent in mice with pulmonary fibrosis [10, 11]. Therefore, for the first time we report that SIK2 acts as a pro-fibrotic factor in the progression of pulmonary fibrosis.

CREB has a wide tissue distribution and plays a pivotal role in depression, metabolic homeostasis and tumorigenesis [7, 21–23]. The effects of SIK2 inactivation were likely due to heightened CRTC2-mediated signaling at CREB. Supporting this conclusion, we observed that SIK2 inactivation increased CRTC2 levels in HFLs (Figs. 1B, 3B) and the anti-fibrotic actions of SIK2 inhibitor ARN-3236 were blocked by the CREB antagonists 666-15. In accordance with our findings, Liu et al. [24] showed that cAMP-mediated activation of CREB DNA binding is decreased in the fibroblasts derived from patients with pulmonary fibrosis. Activation of the cAMP/protein kinase A (PKA)/CREB pathway is absolutely required for the anti-fibrotic effects of phosphodiesterase inhibitors and dibutyryl-cAMP [25, 26]. However, Barlow et al. [27] reported that asbestos exposure induces peribronchiolar fibrosis through activation of PKA/ERK1/2/CREB signaling pathway. This discrepancy with present work may be explained by differences in the models of the pulmonary fibrosis and the genetic background of the mice.

The activation of quiescent fibroblasts and differentiation of fibroblasts to myofibroblasts is critical to the development and progression of pulmonary fibrosis. α-SMA is a biomarker of myofibroblasts, and reflects the proliferation of myofibroblasts. In vitro, our data suggested that inactivation of SIK2 either by ARN-3236 or by siRNA markedly inhibited the proliferation and α-SMA expression of HFLs (Fig. 2A, B). In vivo, our results showed that BLM-induced pulmonary lesions and the expression of α-SMA were dose-dependently alleviated by ARN-3236 (Figs. 4E, 5F), supporting the therapeutic effects of this compound in pulmonary fibrosis. α-SMA also induced excessive production of ECM and fibro-genic proteins, including fibronectin and collagen (Figs. 2A, 4D, F), while ARN-3236 could reduce the expression of these protein in a dose-dependent manner. Collectively, these studies demonstrated that inhibition of SIK2 can alleviate pulmonary fibrosis.

## Conclusion

In summary, current studies found that SIK2 and phosphorylated-CRTC2 were expressed at a low basal level in normal lung tissues and quiescent fibroblasts, but increased in fibrotic lung tissues and activated fibroblast. Inhibition of SIK2 by ARN-3236 prevented the fibroblasts differentiation and ECM expression in a CREB-dependent manner. Our results elucidate a previously unexplored role of SIK2 in pulmonary fibrosis through modulation of CRTC2/ CREB signaling pathway. Our data also identify SIK2 inhibitor ARN-3236 as a promising anti-fibrosis agent.

## Data Availability

The datasets used and/or analyzed during the current study are available from the corresponding author on reasonable request.

## Abbreviations

SIK2: Salt-inducible kinase 2
CREB: CAMP response element binding protein
CRTC2: CREB-regulated transcription co-activator 2
IPF: Idiopathic pulmonary fibrosis
ECM: Extracellular matrix components
BLM: Bleomycin
CTGF: Connective tissue growth factor
TGF-β1: Transforming growth factor-β1
PDGF: Platelet derived growth factor
HFLs: Human fetal lung fibroblasts
